# WGCNA and transcriptome profiling reveal hub genes for key development stage seed size/oil content between wild and cultivated soybean

**DOI:** 10.1186/s12864-023-09617-6

**Published:** 2023-08-28

**Authors:** Yanjie Yao, Erhui Xiong, Xuelian Qu, Junfeng Li, Hongli Liu, Leipo Quan, Wenyan Lu, Xuling Zhu, Meiling Chen, Ke Li, Xiaoming Chen, Yun Lian, Weiguo Lu, Dan Zhang, Xinan Zhou, Shanshan Chu, Yongqing Jiao

**Affiliations:** 1 Collaborative Innovation Center of Henan Grain Crops, College of Agronomy, Henan Agricultural University, Zhengzhou, 450046 China; 2 Key Laboratory of Biology and Genetic Improvement of Oil Crops, Ministry of Agriculture and Rural Affairs, Oil Crops Research Institute of Chinese Academy of Agricultural Sciences, Wuhan, 430062 China; 3 Chongqing Academy of Agricultural Sciences, Chongqing, 401329 China; 4 Zhengzhou Subcenter of National Soybean Improvement Center, Key Laboratory of Oil Crops in Huang-Huai Valleys of Ministry of Agriculture, Institute of Industrial Crops, Henan Academy of Agricultural Sciences, Zhengzhou, China

**Keywords:** Soybean, Oil content, Seed size, Transcriptome, WGCNA, Correlation analysis

## Abstract

**Background:**

Soybean is one of the most important oil crops in the world. The domestication of wild soybean has resulted in significant changes in the seed oil content and seed size of cultivated soybeans. To better understand the molecular mechanisms of seed formation and oil content accumulation, WDD01514 (E1), ZYD00463 (E2), and two extreme progenies (E23 and E171) derived from RILs were used for weighted gene coexpression network analysis (WGCNA) combined with transcriptome analysis.

**Results:**

In this study, both seed weight and oil content in E1 and E171 were significantly higher than those in E2 and E23, and 20 DAF and 30 DAF may be key stages of soybean seed oil content accumulation and weight increase. Pathways such as “Photosynthesis”, “Carbon metabolism”, and “Fatty acid metabolism”, were involved in oil content accumulation and grain formation between wild and cultivated soybeans at 20 and 30 DAF according to RNA-seq analysis. A total of 121 oil content accumulation and 189 seed formation candidate genes were screened from differentially expressed genes. WGCNA identified six modules related to seed oil content and seed weight, and 76 candidate genes were screened from modules and network. Among them, 16 genes were used for qRT-PCR and tissue specific expression pattern analysis, and their expression-levels in 33-wild and 23-cultivated soybean varieties were subjected to correlation analysis; some key genes were verified as likely to be involved in oil content accumulation and grain formation.

**Conclusions:**

Overall, these results contribute to an understanding of seed lipid metabolism and seed size during seed development, and identify potential functional genes for improving soybean yield and seed oil quantity.

**Supplementary Information:**

The online version contains supplementary material available at 10.1186/s12864-023-09617-6.

## Background

Soybean (*Glycine max* L. Merr.) is one of the most economically important protein and oil seed crops [1–3]. At present, the main goal of soybean breeding is to increase soybean oil content and yield. With the development of modern genetic methods and biotechnology [2], obtaining information on genetic loci and genes associated with soybean seed oil and yield has become the main research focus of scientists [4].To date, some key genes related to lipid biosynthesis, such as *GmFAD2* [5], *GmFAD3* [6], *GmDof4* and *GmDof11* [7], *GmMYB73* [8], *GmDREBL* [9], *GmLEC2a* [10] and *GmNFYA* [11], have been identified and applied to the genetic improvement of soybean oil. In *GmFAD2-2* mutant seeds, the oleic acid content increased to 65.58%, while the linoleic acid content decreased to 16.08% [5]. In particular, the content of linolenic acid in seeds was reduced to less than 2% in *GmFAD3-2a* mutant seeds [6]. Seed size and weight are closely related to oil content in soybean [12]. Several genes that regulate seed size have also been identified in soybean [3, 13, 14], including *GmST05* [15], *GmGA20OX* [11], *GmPP2C-1* [16], *GmBS1* and *GmBS2* [17], *GmCYP78A10* and *GmCYP78A72* [18, 19], *GmCIF1* [20], *GmWRKY15a* [21], *GmDREBL* [9] and *GmPIP2;9* [22]. For example, *GmST05*, encodes a member of the PEBP family, identified in soybean using a genome-wide association study (GWAS) of over 1800 soybean accessions. Overexpression and knockout of *GmST05* in soybean can significantly increase and decrease seed size, respectively [15]. These genes could be used to improve soybean oil quality and yield through molecular breeding in the future.

In the last decade, RNA-sequencing technology has been used to study the biosynthesis and regulation of soybean seed oil and seed size [11, 23–25]. *GmGA20OX* (*Glyma.07g08950*) and *GmNFYA* (*Glyma.02g47380*), which encode gibberellin 20 oxidase and nuclear factor Y subunit A, were screened by transcriptome analysis from 40 samples of developing soybean seeds in cultivated and wild soybean. Overexpression of *GmGA20OX* and *GmNFYA* in *Arabidopsis thaliana* increased the seed weight and oil content, respectively [11]. For different developmental stages of Jiyu-72, and a total of 11,592, 16,594 and 16,255 differentially expressed genes (DEGs) were screened at 35 days after flowering (DAF), 55 DAF and 65 DAF compared with 15 DAF, respectively. Among them, 24 genes were involved in lipid biosynthesis pathways [23]. In addition, some genes of the plant hormone signalling pathway and transcription factors were identified in the control of seed size through comparative transcriptome analysis of cultivated soybean and local varieties at three developmental stages. Overexpression of *GmCYP78A5* (*Glyma.05g019200*) in Willams82 (W82) significantly increased seed size and weight [24]. In addition, correlation networks are increasingly being used in bioinformatics applications [26, 27]. Weighted gene coexpression network analysis (WGCNA) is a systems biology method for describing the correlation patterns among genes across microarray samples, which can be used for finding modules of highly correlated genes [28]. *GmABI3b* (*Glyma.08g357600*) was reported to be involved in seed oil accumulation [29]. Combined with transcriptome date and WGCNA of Nannong1138-2 at five seed developmental stages, a total of 124 candidate genes and 12 transcription factor genes, such as *GmABI3b*, *GmNFYA* and *GmFAD2-1B*, were identified to be associated with seed oil accumulation [25]. These results have great significance for soybean oil quality and yield improvement.

However, seed size and oil content are quantitative traits, that are involved in a variety of pathways. Screening more key genes is a key scientific issue to facilitate the construction of their molecular networks. In this study, transcriptome analysis and WGCNA were employed to identify DEGs, stage-specific genes, candidate hub gene clusters and regulatory network modules among ZYD00463 (*G. soja*), WDD01514 (*G. max*), and their RIL progeny at two key developmental stages (20 DAF and 30 DAF). Furthermore, the candidate hub genes were also confirmed by real-time PCR and tissue specific expression pattern analysis. Combined with the expression levels of these genes in 33-wild and 23-cultivated soybean varieties, and correlation analysis with oil content and seed weight, some key genes were verified as likely to be involved in oil content accumulation and grain formation. This study provides valuable new information on the regulation of seed size and oil content during seed development.

## Results

### Dynamic changes in seed oil content and seed weight during soybean development

**Fig. 1:**
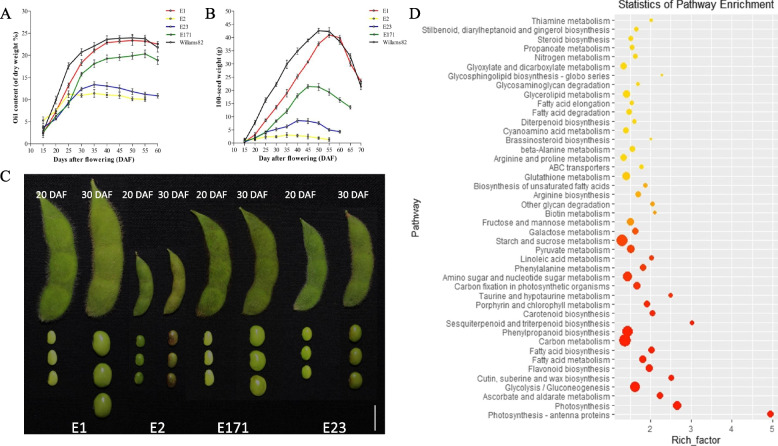
The 20 DAF and 30 DAF are key stage of seeds oil content accumulation/weight increase in soybeans. **A** Dynamic changes of seed oil content during soybean development in E1, E2, E23, E171 and Willams82. **B** Dynamic changes of 100-seed weight during soybean development in E1, E2, E23, E171 and Willams82. **C** The phenotype of seeds at the stage of 20 DAF and 30 DAF with E1, E2, E23 and E171. **D** The KEGG pathway enrichment of G1 between 20 and 30 DAF. Bar, 1 cm

The weight and oil content of seeds are two key factors for soybean quality and yield. Our previous studies have shown that the average seed oil content of WDD01514 was approximately twofold higher than that of ZYD00463 (23.76% vs.11.89%) [30]. Here, the oil content of seeds was studied at different developmental stages using cultivated soybean (E1), wild soybean (E2), W82 and two progenies (E23 and E171) from the E1 and E2 RIL populations (Fig. 1A). The results showed that the oil contents in E1 and E171 were significantly higher than those in E2 and E23, with a tendency to increase first and then stabilize with the development process. The increase rate of oil content was the fastest at the stage of 20–30 days after flowering (DAF) and then slowed after 30 DAF. In addition, due to the rapid growth rate in this period, the completion degree of oil accumulation reached 80% and 90% of E1 and E2 at 30 DAF, respectively. Compared to 20 DAF, the oil content increased 2.51-, 1.47-, 2.22-, and 2.51-fold in E1, E2, E23 and E171 at 30 DAF, respectively, which was much higher than-the increases at 40 DAF vs. 30 DAF (1.25-, 1.01-, 1.05and 1.22fold) (Fig. 1A).

Seed size and weight are closely related to oil content in soybean [12]. To clarify the relationship between oil content and seed weight in E1, E2, E23 and E171, hundred-grain weight (HGW) was also measured in this study (Fig. 1B). Similar to the oil content, the seed weights of E1 and E171 were significantly higher than those of E2 and E23, however, in contrast to the oil content, the HGW first increased and then decreased. Compared to 20 DAF, the HGW increased by 4.25-, 1.66-, 2.65- and 4.51-fold in E1, E2, E23 and E171 at 30 DAF, respectively, which was much higher than the increases at 40 DAF vs. 30 DAF (1.86, 1.17, 1.63 and 2.14-fold) (Fig. 1B). The stage from the20 DAF to 30 DAF may be the key stage for oil content accumulation and seed formation (Fig. 1C). To determine the molecular function in seed weight and oil content during this critical period, the seeds of 20 DAF and 30 DAF stages were used for RNA-seq analysis. To ensure the accuracy of expression, the DEGs were identified using restrictive conditions: FDR < 0.01-, and absolute fold-change≧2.

To identify the key DEGs in E1, E2, E23 and E171 in different developmental stages, we first compared the 20 DAF and 30 DAF stages in E1, E2, E23 and E171. Compared to 20 DAF, a total of 4258, 3313, 2325 and 5790 DEGs were identified at 30 DAF in E1, E2, E23 and E171 (Group 1, G1), respectively. Among them, 1,435 up- and 2,823 downregulated-DEGs were identified in E1 (G1_1); 998 up- and 2,325 downregulated- DEGs were identified in E2 (G1_2); 2,092 up- and 3,878 downregulated-DEGs were identified in E23 (G1_3); and 1,107 up- and 1,872 down-DEGs were identified in E171 (G1_4).

To investigate the pathways associated with the DEGs in E1, E2, E23 and E171, a pathway-based analysis was performed using the KEGG pathway database. As a result, a total of 1787 DEGs were classified into 125 subcategories, among them, “Photosynthesis”, “Glycolysis”, “Ascorbate and aldarate metabolism”, “Starch and sucrose metabolism”, “Carbon metabolism” and “Fatty acid biosynthesis and metabolism” related pathways were significantly enriched (Fig. 1D). These pathways were closely related to grain formation and oil content accumulation, which is consistent with the results of oil content and seed weight from20 DAF to 30 DAF (Fig. 1A, B).

### RNA-seq analysis of oil content accumulation and grain formation at 20 and 30 DAF between wild and cultivated soybeans

**Fig. 2:**
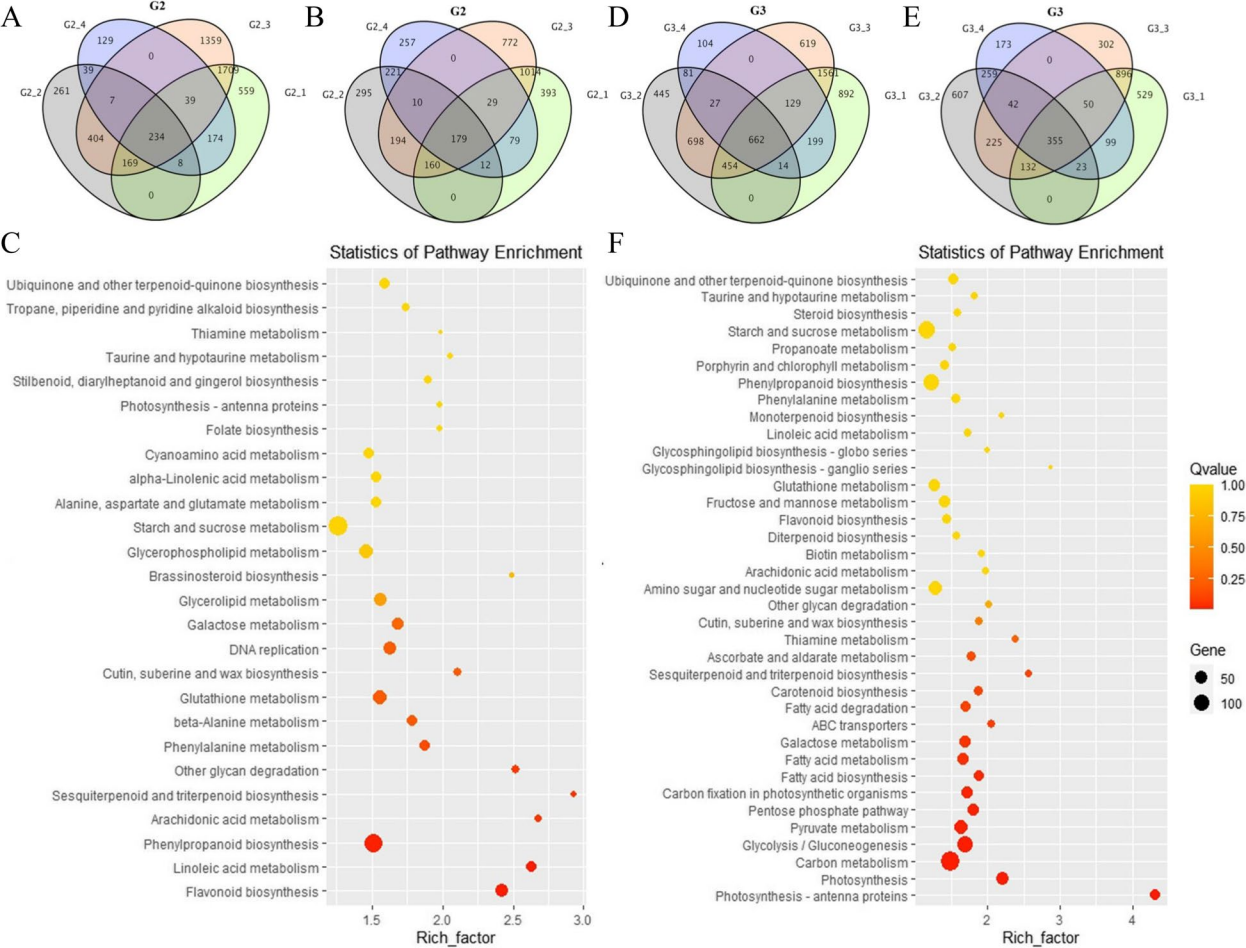
RNA-seq analysis of the oil content accumulation and grain formation between wild and cultivated soybeans at the stage of 20 and 30 DAF. **A** The venn of up-regulated DEGs in G2. **B** The venn of down-regulated DEGs in G2. **C** KEGG enrichment analysis of G2. **D** The venn of up-regulated DEGs in G3. **E** The venn of down-regulated DEGs in G3. **F** KEGG enrichment analysis of G3. The abscissa is the rich factor, and the ordinate is the KEGG path name. The greater the rich factor, the more significant the enrichment level of DEGs in this pathway. The color of the circle represents the *Q* value. The smaller of *Q* value, the more reliable the enrichment significance of DEGs in this pathway. The size of the circle indicates the number of genes enriched in the pathway

To obtain more information on different seed oil content/weight soybean between wild and cultivated soybean, the DEGs of E2 vs. E1 (4758 genes, G2-1), E23 vs. E171 (2193 genes, G2-2), E2 vs. E171 (6279 genes, G2-3) and E23 vs. E1 (1417 genes, G2-4) were screened at 20 DAF, which included 2,892 up- and 1,866 downregulated genes, 1,122 up- and 1,071 downregulated genes, 3921 up- and 2,358 downregulated genes, and 630 up- and 7,87 downregulated genes, respectively (Fig. 2A, B). Among them, a total of 1448 DEGs were classified into 125 subcategories. In contrast to G1, the pathways “Linoleic acid metabolism”, “phenylpropanoid biosynthesis”, “Flavonoid biosynthesis”, “Glutathione metabolism” and “Phenylalanine metabolism”, which are mainly involved in fatty biosynthesis and the stress response, were enriched (Fig. 2C). The results showed that fatty biosynthesis and the stress response may play an important role at 20 DAF between wild and cultivated plants.

In addition, a total of 5,995 DEGs (3911 upregulated and 2804 downregulated), 4,024 DEGs (2381 upregulated and 1,643 downregulated), 6,152 DEGs (4150 upregulated and 2002 downregulated), and 2,217 DEGs (1,216 upregulated and 1,001 down-regulated) were mutually present in the four groups (G3-1, G3-2, G3-3 and G3-4, respectively) at 30 DAF (Fig. 2D, E). The pathways “Photosynthesis”, “Carbon metabolism”, “Glycolysis”, “Pyruvate metabolism”, “Pentose phosphate” and “Fatty acid biosynthesis, degradation and metabolism” were enriched in this group (Fig. 2F). Similar to G1 but different from G2, revealing that nutrient accumulation and fatty acid metabolism played a key role at 30 DAF between wild and cultivated development. These pathways are the basis of material synthesis and the source of raw material power, which contribute to grain formation and oil content in soybean seed development.

**Fig. 3:**
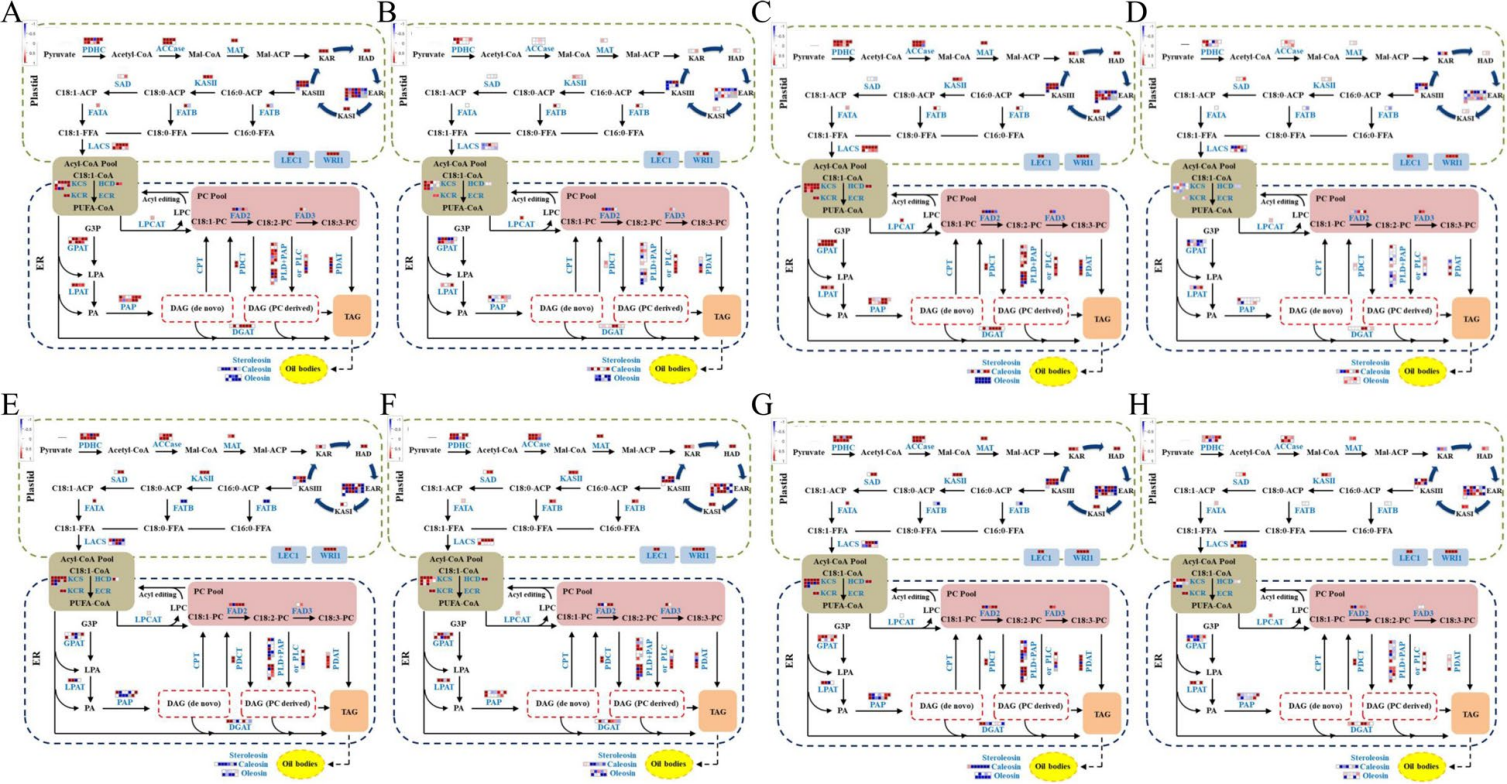
The expression of DEGs associated with lipid metabolic pathways in G2 and G3. **A**-**D** The expression of DEGs associated with lipid metabolic pathways in G2-1, G2-2, G2-3 and G2-4 between wild and cultivated soybeans at the stage of 20 DAF. **E**–**H** The expression of DEGs associated with lipid metabolic pathways in G3-1, G3-2, G3-3 and G3-4 between wild and cultivated soybeans at the stage of 30 DAF. Every square represents a gene in lipid metabolic pathways. The color represents the log_2_ (FC) of the gene with down-regulation in blue while up-regulation in red

Furthermore, to better understand the role of these DEGs in soybean oil content accumulation, a total of 121 (Table S3) candidate genes were screened from the DEGs (G2 and G3) for MapMan software analysis. The results showed that these genes were significantly regulated in lipid metabolism pathway between wild and cultivated soybeans at 20 DAF (Fig. 3A-D) and 30 DAF (Fig. 3E-H) and were involved in almost biochemical reactions in the pathway. Compared to high oil content wild soybeans, genes such as Enoyl-ACP reductase (EAR: *Glyma.07g258200*, *Glyma.19g238900*, *Glyma.06g162800* and *Glyma.16g181300*) in the plastid, and phosphatidate phosphatase (PAP: *Glyma.20g121200*, *Glyma.10g270100*, *Glyma.17g242900* and *Glyma.17g139100*) and phospholipase D (PLD, *Glyma.03g018900* and *Glyma.20g238000*) in the ER were significantly downregulated, which may reduce the lipid or oil body content. Interestingly, the pyruvate dehydrogenase complex (PDHC) genes (*Glyma.01g081900*, *Glyma.01g038000*, *Glyma.13g119800*, *Glyma.20g114500*, *Glyma.17g040500* and *Glyma.02g026800*) of first reaction in plastid and 3-ketoacyl-CoA synthase (KCS) genes (*Glyma.06g058500*, *Glyma.10g179400*, *Glyma.06g012500*, *Glyma.06g214800*, *Glyma.04g149300* and *Glyma.20g115500*) of the first reaction in the ER were significantly upregulated, which may be influenced by feedback regulation. These altered genes affect oil body synthesis and ultimately lead to an increase in oil content.

**Fig. 4:**
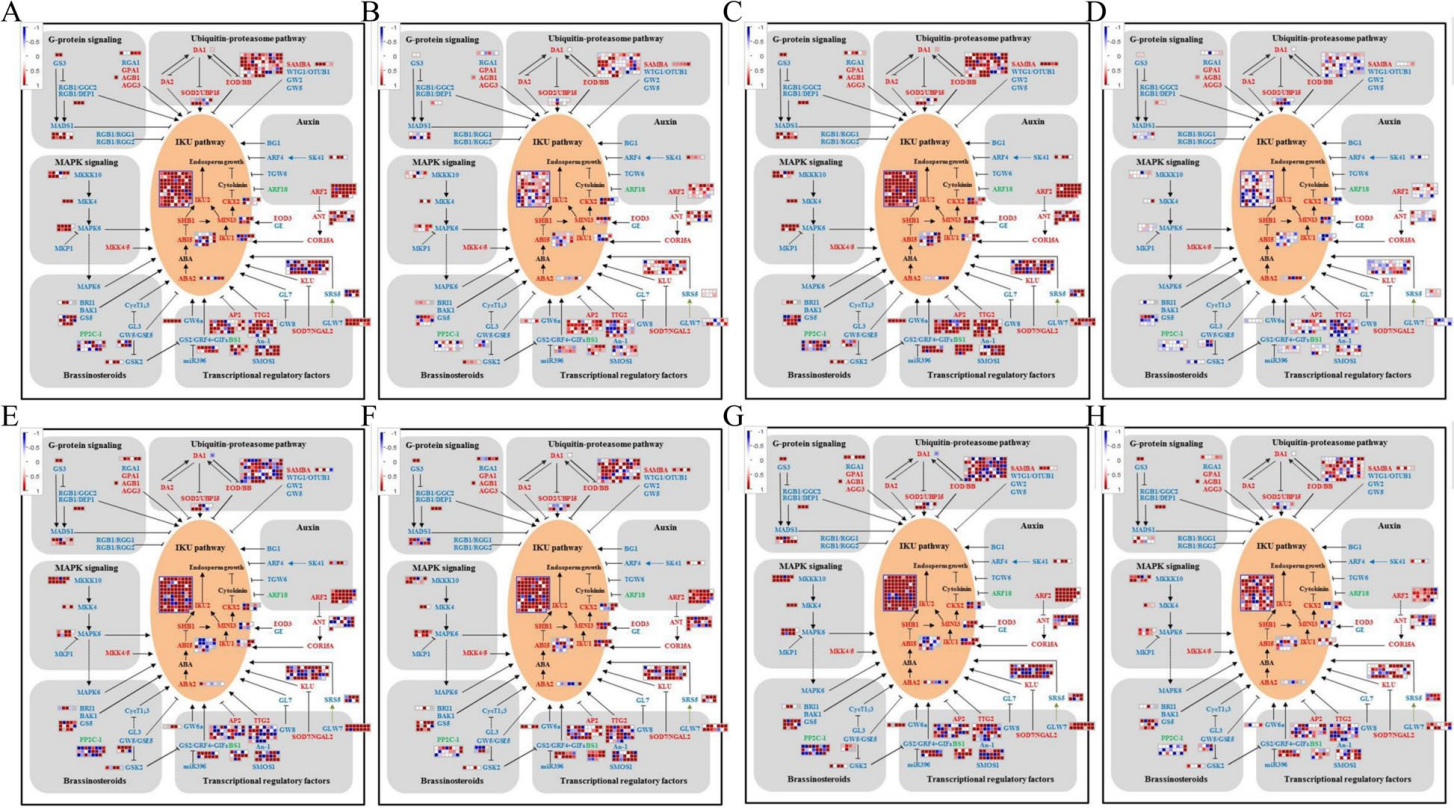
Network of seed size control with different expression of DEGs in G2 and G3. **A**-**D** The expression of DEGs associated with seed size related pathways in G2-1, G2-2, G2-3 and G2-4 between wild and cultivated soybeans at the stage of 20 DAF. **E**–**H** The expression of DEGs associated with seed size related pathways in G3-1, G3-2, G3-3 and G3-4 between wild and cultivated soybeans at the stage of 30 DAF. Every square represents a gene in regulation of seed size. The color represents the log_2_ (FC) of the gene with down-regulation in blue while up-regulation in red

In addition, a total of 189 (Table S4) candidate genes related to grain formation were screened from DEGs (G2 and G3) for MapMan software analysis. These genes were also significantly regulated in the grain formation pathway between wild and cultivated soybeans at 20 DAF (Fig. 4A-D) and 30 DAF (Fig. 4E-H). Compared to the smaller size seeds of wild soybeans, some genes such as phosphatase 2C (*Glyma.08g081400*, *Glyma.11g018000* and *Glyma.06g238200*), E3 ubiquitin ligase (*Glyma.09g256800*, *Glyma.12g081100* and *Glyma.18g013800*), LRR receptor kinase (*Glyma.16g064900* and *Glyma.15g021600*), mitogen-activated protein kinase kinase kinase (*Glyma.14g080100* and *Glyma.20g142900*), cytochrome P450 (*Glyma.20g114200* and *Glyma.07g220400*), and many transcription factors (*Glyma.08g142400*, *Glyma.05g228100*, *Glyma.05g245400*, *Glyma.19g094100* and *Glyma.04g041200*) were significantly changed, which affected grain formation and decreased grain weight.

### Weighted gene coexpression network analysis (WGCNA)

**Fig. 5:**
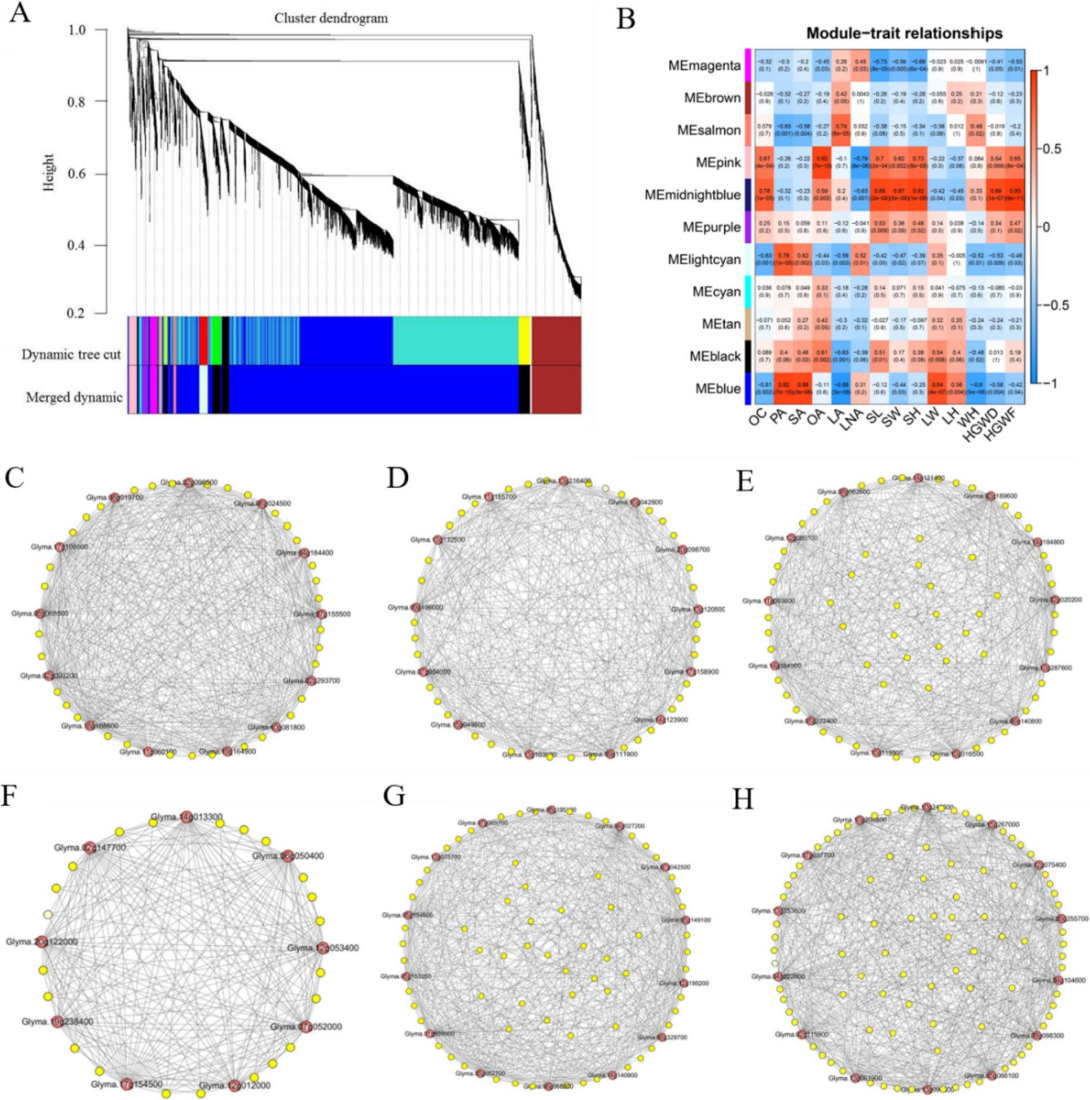
Weighted gene co-expression network analysis (WGCNA). **A** Gene cluster dendrograms and module detecting. **B** Heat map of module-trait correlation. **C**-**H** Gene co-expression network analysis in six modules of magenta, salmon, pink, midnight, lightcyan and blue. The abscissa represents different traits, the vertical ordinate represents different modules. Red color of each box represents the positive correlation between module and trait; blue color represents the negative relationships between module and trait. Candidate hub genes are shown in red. Each node represents a gene, and each edge represents the co-expression correlation between genes

WGCNA was used to screen important modules, build a gene coexpression network and filter the hub genes [28]. Modules were defined as clusters of highly interconnected genes, and genes within the same cluster had high correlation coefficients among them. Combined with the transcriptome and phenotype data for24 samples, WGCNA of the modules was conducted by R software (Fig. 5A). Here, a total of 11 distinct modules (labelled with different colours) were identified; among them (Fig. 5B), six of 11 coexpression modules were significantly correlated with traits. For example, the magenta module was significantly negatively correlated with seed length (SL, correlation coefficient -0.73), the salmon module was significantly positively correlated with linoleic acid (LA, correlation coefficient 0.73), and midnightblue module was significantly positively correlated with seed width SW (correlation coefficient 0.87), SH (correlation coefficient 0.92), HGWD (correlation coefficient 0.86) and HGWF (correlation coefficient 0.93) (Fig. 5B).

WGCNA can also be employed to construct gene networks, in which each node represents a gene and the connecting lines (edges) between genes represent coexpression correlations. Highly connected genes (Hub genes) are those that show the most connections in the network or high kME value in module. In this study, a total of 69 hub genes with the highest kME values were screened in the six modules according to the kME value (Table S5). Furthermore, the gene coexpression network was also constructed by Cytoscape software, and 76 hub genes were identified according to connections (Fig. 5C-H). The results showed that some hub genes, such as omega-6 fatty acid desaturase (FAD2-2, *Glyma.09g111900*), glycerol-3-phosphate acyltransferase 6 (GPAT6, *Glyma.10g119900*), glycerol-3-phosphate acyltransferase 8 (GPAT8, *Glyma.07g069700*) and stearoyl-ACP desaturase (SAD, *Glyma.14g121400*), gibberellin-2-oxidase (GA2OX, *Glyma.13g287600*), ABA receptor PYL4 (*Glyma.07g155500*) and transcription factors (*Glyma.06g068800*, *Glyma.07g054000*, *Glyma.09g098300*), were involved in lipid metabolism and seed formation. These genes may play a key role in grain formation and oil content in soybean seed development.

### qRT-PCR and tissue-specific expression analysis of key DEGs

qRT-PCR is usually used to confirm the credibility of RNA-Seq data. In this study, a total of 16 genes associated with lipid metabolism and the regulation of seed size were screened by transcriptome analysis and WGCNA for qRT‒PCR analysis (Figs. S1, S2 and S3). These genes were quantized and analysed with G1-1 (Fig. S1A), G1-2 (Fig. S1B), G1-3 (Fig. S1C), G1-4 (Fig. S1D), G2-1 (Fig. S2A), G2-2 (Fig. S2B), G2-3 (Fig. S2C), G2-4 (Fig. S2D), G3-1 (Fig. S3A), G3-2 (Fig. S3B), G3-3 (Fig. S3C) and G3-4 (Fig. S3D). Compared to the RNA-seq results, the qRT‒PCR expression pattern of 167 of 192 genes (approximately 88.54%) were consistent with the RNA-seq data. The results indicated that the RNA-seq date were highly reliable.

**Fig. 6:**
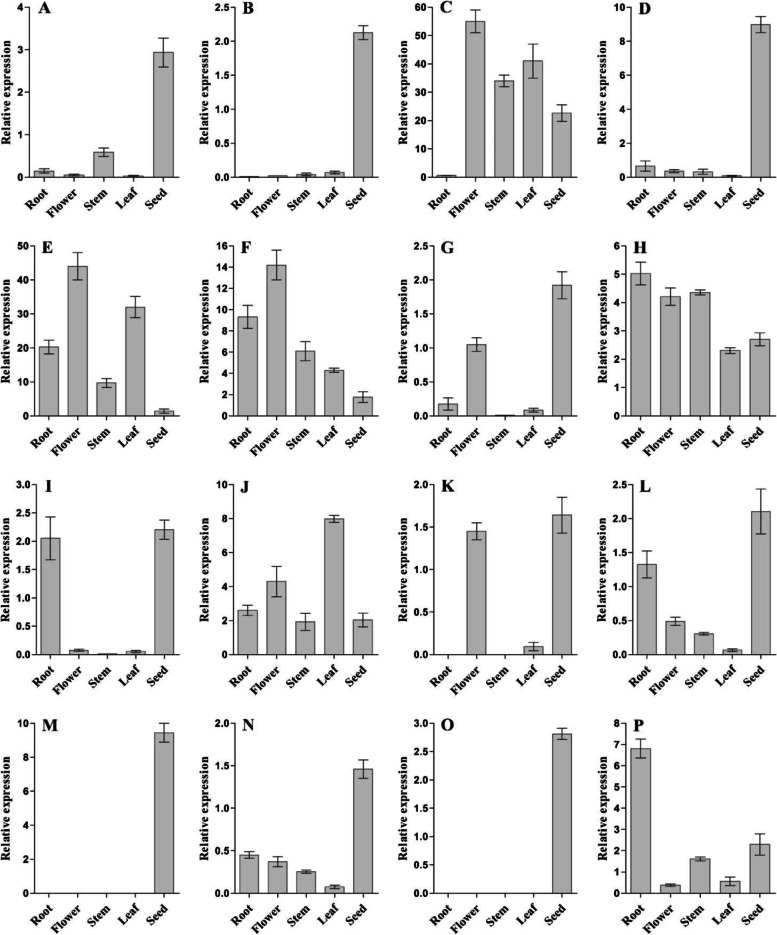
Tissue specific expression of candidate genes using qRT-PCR. **A**-**D** The tissue specific expression of *Glyma.03g189600*, *Glyma.06g050400*, *Glyma.06g068800* and *Glyma.07g037700* at the stage of 30 DAF in W82; **E**–**H** The tissue specific expression of *Glyma.07g06970*0, *Glyma.09g098300*, *Glyma.09g111900* and *Glyma.09g255700* at the stage of 30 DAF in W82; **I**-**L** The tissue specific expression of *Glyma.10g119900*, *Glyma.13g253600*, *Glyma.13g287600* and *Glyma.14g123900* at the stage of 30 DAF in W82; **M**-**P** The tissue specific expression of *Glyma.14g184900*, *Glyma.17g154500*, *Glyma.19g164900* and *Glyma.20g122000* at the stage of 30 DAF in W82, respectively. The abscissa represents the root, stem, leaf, flower and seed organ of soybean

In addition, tissue-specific expression of 16 genes was analysed in roots, stems, leaf, flowers and seeds at 30 DAF in W82 (Fig. 6). The results showed that the *Glyma.03g189600*, *Glyma.06g050400*, *Glyma.07g037700*, *Glyma.14g184900* and *Glyma.19g164900* genes were specifically expressed in developing soybean seeds (Fig. 6A, B, D and M), *Glyma.09g111900* and *Glyma.13g287600* were specifically expressed in flowers and seeds (Fig. 6G, K), and *Glyma.10g119900* was specifically expressed in flowers and seeds (Fig. 6I).In contrast, *Glyma.10g119900* was expressed at lower levels in and highly expressed in roots, flowers, stems and leaf (Fig. 6E).

### Expression levels of candidate key genes between 33-wild and 23-cultivated soybean varieties

**Fig. 7:**
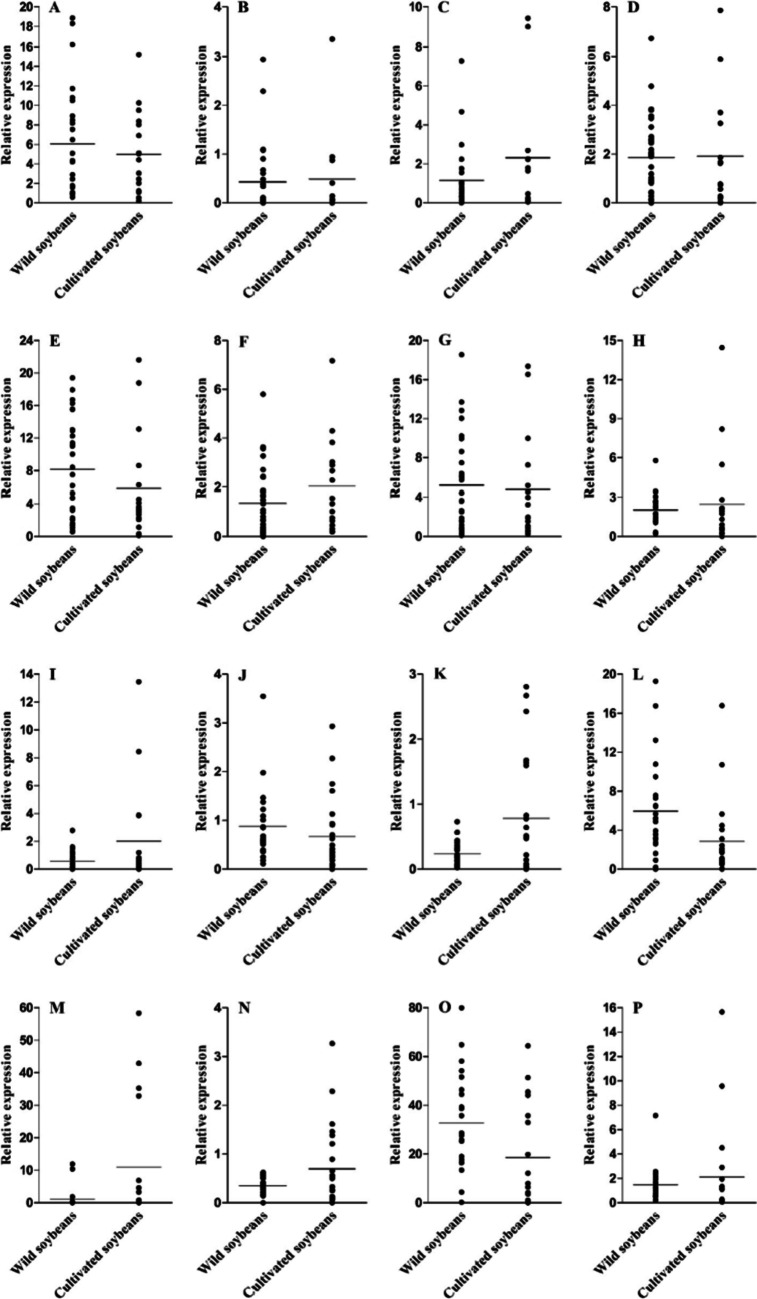
The relative expression of candidate genes between wild and cultivated soybean seed. **A**-**D** The gene relative expression level of *Glyma.03g189600*, *Glyma.06g050400*, *Glyma.06g068800* and *Glyma.07g037700* between wild and cultivated soybean seed; **E**–**H** The gene relative expression level of Glym*a.07g06970*0, *Glyma.09g098300*, *Glyma.09g111900* and *Glyma.09g255700* between wild and cultivated soybean seed; **I**-**L** The gene relative expression level of *Glyma.10g119900*, *Glyma.13g253600*, *Glyma.13g287600* and *Glyma.14g123900* between wild and cultivated soybean seed; **M**-**P** The gene relative expression level of *Glyma.14g184900*, *Glyma.17g154500*, *Glyma.19g164900* and *Glyma.20g122000* between wild and cultivated soybean seed, respectively. The abscissa represents seeds of wild and cultivated soybean

To further explore the functional and evolutionary significance of these genes between wild and cultivated varieties, we compared the expression levels of these 16 genes in wild and cultivated soybean seed (Fig. 7). The results showed that the average expression levels of *Glyma.06g068800*, *Glyma.09g098300*, *Glyma.10g119900*, *Glyma.13g287600*, *Glyma.14g184900* and *Glyma.17g154500* in cultivated soybean seeds were higher than those in wild soybean seeds (Fig. 7C, F, G, K, M and N), suggesting that these genes may have been fixed under artificial selection during soybean domestication. In addition, compared to wild soybean, the average expression levels of *Glyma.03g189600*, *Glyma.07g069700*, *Glyma.13g253600*, *Glyma.14g123900* and *Glyma.19g164900* were decreased in cultivated soybean varieties (Fig. 7A, E, J, L and O). The expression of other genes did not change significantly between wild soybean and cultivated soybean. Therefore, these genes may have important biological significance between wild soybean and cultivated soybean.

### The correlation between the expression of candidate key genes and grain weight/oil content

**Fig. 8:**
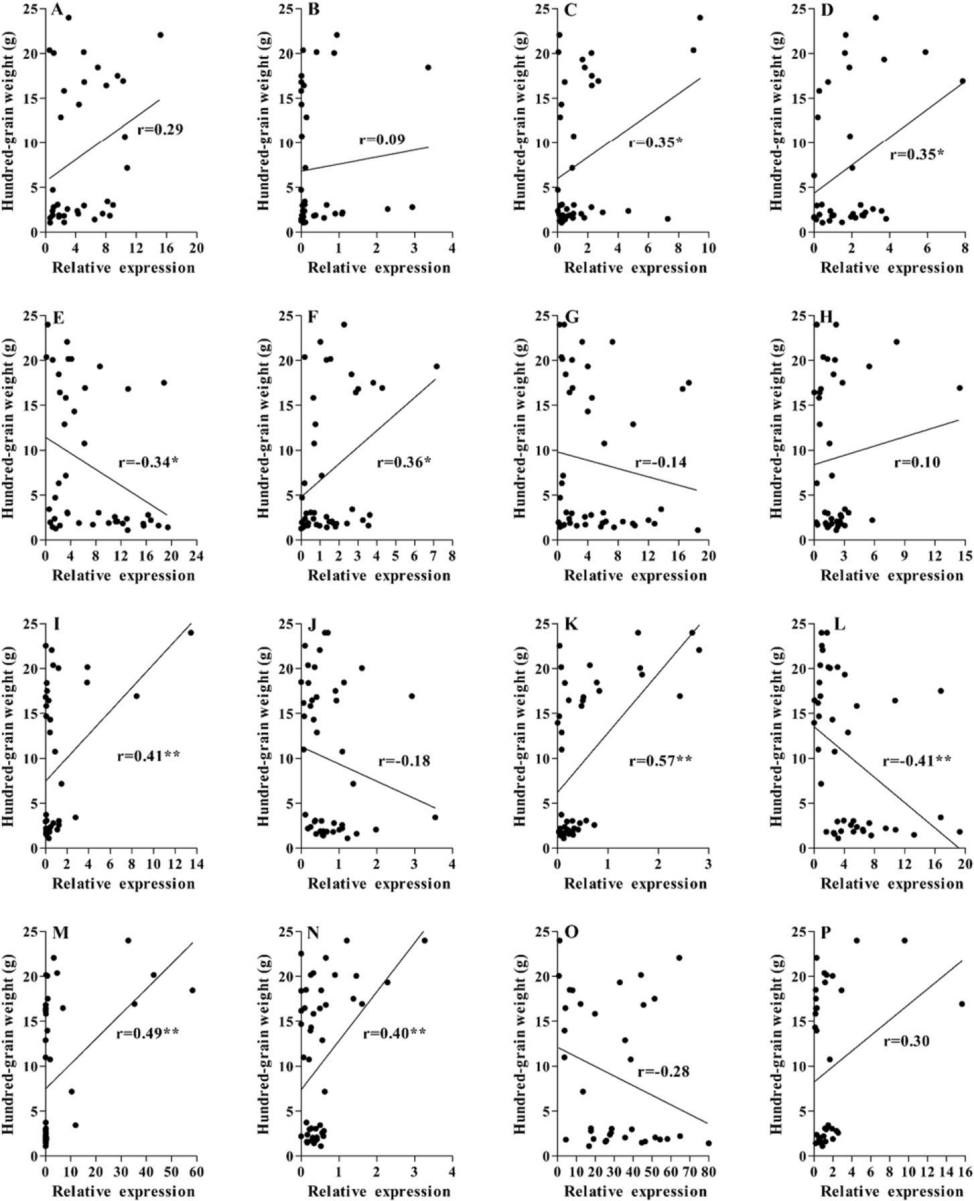
Correlation analysis between candidate gene expression level and seed oil content. **A**-**D** The correlation between oil content and relative expression level of *Glyma.03g189600*, *Glyma.06g050400*, *Glyma.06g068800* and *Glyma.07g037700*; **E**–**H** The *Glyma.07g06970*0, *Glyma.09g098300*, *Glyma.09g111900* and *Glyma.09g255700*; **I**-**L** The correlation between oil content and relative expression level of *Glyma.10g119900*, *Glyma.13g253600*, *Glyma.13g287600* and *Glyma.14g123900*; **M**-**P** The correlation between oil content and relative expression level of *Glyma.14g184900*, *Glyma.17g154500*, *Glyma.19g164900* and *Glyma.20g122000*, respectively*.* The abscissa represents the relative expression level of gene calculated by 2.^−ΔΔCT^; The ordinate represents seed oil content (%). * represents significance of correlation at *P* < 0.05; ** represents significance of correlation at *P* < 0.01

**Fig. 9:**
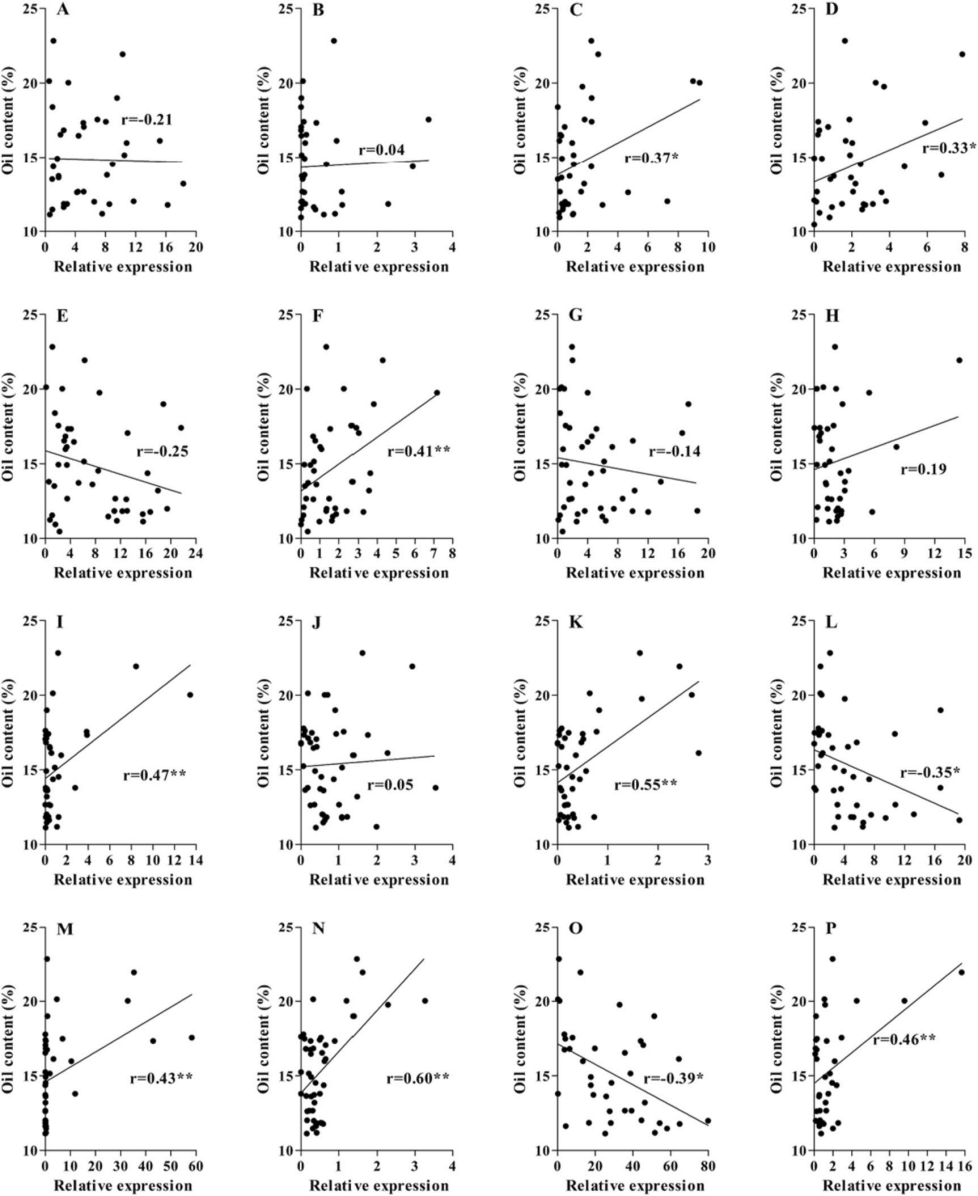
Correlation analysis between candidate gene expression level and hundred-grain weight. **A**-**D** The correlation between hundred-grain weight and relative expression level of *Glyma.03g189600*, *Glyma.06g050400*, *Glyma.06g068800* and *Glyma.07g037700*; **E**–**H** The correlation between hundred-grain weight and relative expression level of *Glyma.07g06970*0, *Glyma.09g098300*, *Glyma.09g111900* and *Glyma.09g255700*; **I**-**L** The correlation between hundred-grain weight and relative expression level of *Glyma.10g119900*, *Glyma.13g253600*, *Glyma.13g287600* and *Glyma.14g123900*; **M**-**P** The correlation between hundred-grain weight and relative expression level of *Glyma.14g184900*, *Glyma.17g154500*, *Glyma.19g164900* and *Glyma.20g122000*, respectively*.* The abscissa represents the relative expression level of gene calculated by 2.^−ΔΔCT^; The ordinate represents hundred-grain weight (g). * represents significance of correlation at *P* < 0.05; ** represents significance of correlation at *P* < 0.01

Furthermore, the correlation between the expression of candidate key genes and grain weight/oil content was also analysed. The results showed that eight genes, including *Glyma.06g068800*, *Glyma.07g037700*, *Glyma.09g098300*, *Glyma.10g119900*, *Glyma.13g287600*, *Glyma.14g184900*, *Glyma.17g154500* and *Glyma.20g122000*, were significantly positively correlated with seed oil content (Fig. 8), while the expression levels of *Glyma.14g123900* and *Glyma.19g164900* were significantly negatively correlated with seed oil content. Meanwhile, for grain weight, the expression levels of *Glyma.06g068800*, *Glyma.07g037700*, *Glyma.09g098300*, *Glyma.10g119900*, *Glyma.13g287600*, *Glyma.14g184900* and *Glyma.17g154500* were significantly positively correlated with hundred-grain weight. The expression levels of *Glyma.07g069700* and *Glyma.14g123900* were significantly negatively correlated with hundred-grain weight (Fig. 9). These genes were involved in grain development, influencing grain size and oil accumulation.

## Discussion

### 20 DAF and 30 DAF are key stages of seed oil content accumulation/weight increase in soybeans

Seed development can be divided into endosperm development, cell division, embryo and cotyledon differentiation, embryo development, seed dehydration and carbohydrate accumulation [31]. In general, the cell differentiation of soybean seed is completed at 20 DAF to 25 DAF. At this stage, the cells have greatly proliferated and the seed size is constantly increasing, while the oil, protein and carbohydrates in the seed are continuously generated and accumulate from 25 to 60 DAF [32]. In this study, the results of seed weight and oil content analysis during development showed that 20 DAF and 30 DAF were the key stages of soybean seed oil content accumulation/weight increase in cultivated soybeans and wild soybeans (Fig. 1A, B). RNA-seq and KEGG pathway analysis showed that the pathways of “Photosynthesis”, “Glycolysis”, “Starch and sucrose metabolism”, “Carbon metabolism” and “Fatty acid biosynthesis and metabolism” were significantly enriched from 20 to 30 DAF (Fig. 1D). At 20 DAF and 30 DAF, developing seeds are still undergoing photosynthesis, which can provide intermediates for the synthesis of substances during seed development [33]. Glycolysis plays a very important role in both carbon metabolism and plant development, converting sucrose produced by photosynthesis into precursors of protein and fatty acid biosynthesis [34].Therefore, these pathways were closely related to grain formation and oil content, which is consistent with the results of oil content and seed weight.

To obtain more information on oil content/weight soybean in wild and cultivated soybean seeds, the DEGs between wild and cultivated seeds were screened and analysed by RNA-seq and pathway enrichment at of 20 DAF and 30 DAF, respectively (Fig. 2). The results showed that the pathways “Linoleic acid metabolism”, “phenylpropanoid biosynthesis”, “Phenylalanine metabolism”, “Photosynthesis”, “Carbon metabolism”, “Glycolysis”, “Pyruvate metabolism”, “Pentose phosphate” and “Fatty acid biosynthesis, degradation and metabolism” were enriched at 20 and 30 DAF between wild and cultivated soybeans. Among them, carbon metabolism, photosynthesis, pyruvate metabolism and glycolysis can provide carbon sources for fatty acid biosynthesis [35]. Pyruvate is converted by glucose through the glycolysis pathway, and then catalysed by pyruvate dehydrogenase to acetyl-CoA, which is directly used for the de novo synthesis of fatty acids [36]. In addition, pyruvate metabolism, glycolysis, the pentose phosphate pathway, and photosynthesis can provide reducing power and ATP for biochemical reactions [37, 38], which play a key role in fatty acid biosynthesis. Therefore, these pathways play an important role in regulating the accumulation of seed oil. Seed size and weight are closely related to oil content in soybean [11, 29]. Multiple signalling pathways are involved in the regulation of seed size, including photosynthesis, carbon metabolism, histone methylation, hormone signalling pathway, ubiquitin protease pathway and MKKK pathway [3, 27, 39, 40]. These pathways regulate seed size by controlling embryo and endosperm development and the proliferation and growth of seed coat or hull cells. For example, the soybean seed weight QTL *PP2C-1*, encoding phosphatase 2C, controls seed size by regulating seed coat cell size by interacting with the GmBZR1 transcription factor in the brassinosteroid (BR) signalling pathway [16]. In this study, some biological processes associated with the above signalling pathways were significantly enriched in the KEGG analysis and play an important role in seed size formation and oil content accumulation. The results were consistent with the seed size/weight and oil content in wild and cultivated soybeans.

### Key genes associated with seed size and oil content between wild and cultivated soybeans at 20 and 30 DAF

The seed oil content and seed weight of soybean are domestication-related traits (DRTs), which are selected during domestication under intense human-directed selection, and the genes controlling DRTs are also strongly selected and fixed [41]. In general, the expression level of genes controlling DRTs, such as *GmNFYA*, *GmGA20OX*, *GmWRKY15a* and *GmOLEO1* [11, 21, 42] were higher in cultivated soybean than in wild soybean. In this study, some candidate genes involved in seed size and oil content were screened by transcriptome analysis. For example, *Glyma.05g221100* encodes acetyl-CoA carboxylase, which catalyses the production of malonyl CoA from acetyl-CoA as arate-limiting enzyme of fatty acid synthesis [43]. *Glyma.05g151200* and *Glyma.05g216600* encodes long chain acyl-CoA synthetase (LACS), which catalyces free fatty acids to produce acyl-CoA [44]. *Glyma.13g118300* encodes diacylglycerol acyltransferase (DGAT), which catalyses the final step of triacylglycerol biosynthesis [45]. *Glyma.10g189900*, *Glyma.14g125500* and *Glyma.19G004800* encode the oil body proteins caleosin and oleosin, which play an important role in stabilizing oil body structure and lipid accumulation [42]. The results showed that overexpression of these genes in different species could increase the oil content. In addition, some candidate genes play an important role in determining oil content and fatty acid composition, such as stearoyl-ACP desaturase (SAD, *Glyma.14g121400*) and fatty acid desaturase 2 (FAD2, *Glyma.09g111900*). At present, one of the most important goals of soybean improvement is to increase monounsaturated fatty acids and reduce polyunsaturated fatty acids. These genes can be used to improve the quality of soybean oil. In addition, *Glyma.06g238200*, *Glyma.08g081400* and *Glyma.11g018000* encode phosphatase 2C (PP2C). PP2C interacts with GmBZR1 in the BR signalling pathway and dephosphorylates GmBZR1. Thus, it controls seed size by regulating the BR signalling pathway [16].

Furthermore, a total of 16 candidate genes involved in seed size and oil content were screened by transcriptome, WGCNA and bioinformatics analysis, and then validated by qRT-PCR, tissue-specific expression and correlation analysis. The average expression level of six out of 16 genes in cultivated soybean grains was higher than that in wild soybean, including *Glyma.06g068800*, *Glyma.09g098300*, *Glyma.10g119900*, *Glyma.13g287600*, *Glyma.14g184900* and *Glyma.17g154500*, which may be affected by artificial selection during soybean domestication [46]. Correlation analysis also showed that these six genes were significantly positively correlated with oil content (Fig. 8) and hundred-grain weight (Fig. 9). The ethylene response transcription factor may be playing an important role in oil content accumulation [47, 48]. For exampleGmWRI1a, was positively regulates seed oil accumulation in soybean, the oil content was increased in *GmWRI1a* overexpressing transgenic plant seeds, especially in oleic acid and linoleic acid [47]. *Glyma.06g068800* encodes the ethylene response transcription factor AP2/EREBP and is a homologue of SHINE 2 in *Arabidopsis thaliana*. SHINE 2 has been shown to regulate epidermal wax and cuticle synthesis in *Arabidopsis*, which has been demonstrated in soybeans [49]. The wax and cuticle are mainly distributed on the surface of the epidermal cells of aerial tissues. Here, the tissue-specific expression analysis showed that *Glyma.06g068800* was specifically expressed in all organs except the roots (Fig. 6). So, we speculate that *Glyma.06g068800* is mainly involved in the synthesis of wax and cuticle in epidermal cells.

The MYB transcription factor, *Glyma.07g037700*, was expressed specifically in seeds and positively correlated with oil content and hundred-grain weight. The seed oil content, seed length and weight were increased when *GmMYB73* was overexpressed in *Arabidopsis* [8]. In addition, *Glyma.13g287600*, encodes a GA2OX, is expressed specifically in flowers and seeds [11]. The results of tissue specific expression, correlation analysis, and expression level analysis between wild and cultivated soybean were consistent with previous studies of *GA20OX* (*Glyma.07g08950*) in soybean seed weight [11]. Furthermore, the c gibberellin content, grain yield and fruit weight were decreased when GA2OX was exogenously expressed in rice and tomato [50, 51]. *Glyma.10g119900*, encoding glycerol-3-phosphate acyltransferase (GPAT), was specifically expressed in roots and seeds and catalyses acylation at the *sn-1* position of glycerol-3-phosphate to produce lysophosphatidic acid (LPA), which is an important intermediate for the formation of different acyl-lipids. Previous studies have shown that GPAT4 is highly expressed in seed coats and roots, and can catalyse the synthesis of suberin [52], which is consistent with the tissue-specific expression in this study. However, the relationship between GPAT and seed weight has not been reported. In addition, *Glyma.14g184900* was specifically expressed in seeds and is located in the QTL *qLNA14_1* of linolenic acid [30]. However, this gene has no relevant functional annotation information in the soybean reference genome, and its specific biological function needs to be further verified.

## Conclusion

To identify the candidate genes and explore the mechanisms of soybean seed lipid metabolism and the regulation of seed size, comparative transcriptome sequencing was studied among two parents and two progenies with different oil content/seed weight at two developmental stages. Combined with transcriptome, WGCNA, bioinformatics, qRT-PCR, tissue-specific expression and correlation analyses, key genes such as *Glyma.06g068800*, *Glyma.09g098300*, *Glyma.10g119900*, *Glyma.13g287600*, *Glyma.14g184900* and *Glyma.17g154500*, which may be involved in seed formation and oil content accumulation, were screened and verified. Overall, these results contribute to an understanding of seed lipid metabolism and seed size during seed development, and provide potential functional genes for improving soybean yield and seed oil quantity.

## Methods

### Plant materials

The parents of WDD01514 (*G. max*) and ZYD00463 (*G. soja*) named as E1 and E2 were identified by Yongqing Jiao and preserved by Henan Agricultural University (Zhengzhou, China) and the Oil Crops Research Institute of the Chinese Academy of Agricultural Sciences (Wuhan, China). The material of E23 and E171 were selected from RIL population derived from ZYD00463 and WDD01514. There was a great difference in the seed oil content and seed weight among the four materials (Table S1). All four soybeans were planted at experimental stations in Hubei Province, Wuhan (N30°35', E114°33'). The developing seed was label after flowering in July every five days. We hand-collected the four soybean developing seed at 20 and 30 days after flowering (20 DAF and 30 DAF) considered as E and M development stage (three biological repetitions of each sample), and total 24 samples for transcriptome sequencing. In addition, the developing seed of 33-wild soybeans and 23-cultivated soybeans at 30 DAF were planted and collected for qRT-PCR analysis. Each was divided into two, one was used to determine traits index associated with seed oil and seed weight, the other was frozen with liquid nitrogen and stored at -80℃ for RNA extraction.

### Total RNA extraction, cDNA library construction and transcriptome sequencing

Total RNA was extracted from the developing seeds collected at 20 and 30 DAF, respectively, using a TIANGEN RNA Prep Pure Plant kit (Tiangen Biotech Co. Ltd, Beijing, China), and purified with the Dynabeads Oligo (dT) 25 kit (Life, USA). The cDNA library was constructed by using a NEBNext Ultra RNA Library Prep Kit (NET, USA), the quality of retrieved cDNA was checked using the Agilent Bioanalyzer 2100 (RNA Nano Chip, Agilent), and it was sequenced using an Illumina HiSeq 4000 paired-end sequencing system.

### Differentially expressed genes (DEGs) analysis

We used FPKM values (Fragments per kilobase of transcript per million fragments mapped) as an indicator of transcript or gene expression levels [53]. FPKM normalizes counts of short sequences by read depth and transcript length. We applied a pairwise transcriptome comparison between the control and experimental groups. Differentially expressed genes (DEGs) was performed using EdgeR (Robinson et al., 2010), and genes with a false discovery rate (FDR) < 0.01 and a fold change (FC) ≥ 2.0 were considered significantly differentially expressed between the control and experimental groups.

### Kyoto encyclopedia of genes and genomes (KEGG) pathway enrichment analysis

The DEGs were annotated in the KEGG database (http://www.genome.jp/kegg) [54, 55], which is an important public database of metabolic pathways and functions for gene products. Using the KEGG pathway as a unit, we compared pathways that were significantly associated with DEGs with the genomic background by computing a P-value using the hypergeometric distribution and FDR for multiple testing (*P* < 0.05). Meanwhile, MapMan software was used to make the expression level of DEGs in the metabolic pathway more intuitive [56].

### Weighted gene co-expression network analysis (WGCNA)

As input files for FPKM values of all genes and phenotype data of 24 samples, weighted gene co-expression network analysis (WGCNA) package in R software (Version 3.4.4) was used to identify modules of highly correlated genes (Langfelder and Horvath 2008), and the results were visualized using Cystoscope software (Version 3.6.1). We used pickSoftThreshold function to calculate the soft power, and finally construct the co-expression network at soft power (β = 10). We selected 0.25 as mergeCutHeight parameter and merged different modules with similarity more than 75%, and then genes with similar expression patterns were merged into the same module. We calculated the kME (Module Eigengene) value, which is used to evaluate the effective connectivity between hub genes. In this study, we selected modules with kME > 0.7. These genes can better represent the expression trend of the whole module. Finally, we determined the hub genes in module according to the kME value and the degree of the genes in the network.

### Quantitative real-time PCR (qRT-PCR) analysis

Total RNA was isolated from frozen developing seeds (30 DAF) of 33 wild soybeans and 23 cultivated soybeans. cDNA synthesis was prepared with 1 μg total RNA using HiScript II QRT SuperMix Kit (Vazyme, China) according to manufacturer’s instructions. qRT-PCR was performed on three biological replicates. The soybean *GmUKN1*, *GmTUB4* and *GmACT11* genes were used to normalize gene expressions. Relative expression levels of target genes were calculated by the 2^−ΔΔCt^ comparative threshold cycle (Ct) method [57]. All gene-specific primers in this paper were showed in (Table S2).

### Supplementary Information

**Additional file 1: Table S1.** Statistics of traits associated with soybean seed oil and seed weight for RNA-seq sample. SL: seed length; SW: seed width; SH: seed height; HGW: hundred-grain weight ; OC: oil content; OA: oleic acid; LNA: linolenic acid. The values associated with seed size and oil were continuously measured from 2015 to 2017. The data is presented in the form of mean ± standard deviation (SD). **Table S2.** Information of all primer sequence in qRT-PCR. **Table S3.** The candidate genes involved in lipid metabolism. **Table S4.** The candidate genes involved in regulation of seed size. **Table S5.** Candidate hub genes in six modules according to kME value. **Fig. S1.** qRT-PCR validation of candidate genes in G1 group. (A) The fold change (FC) of gene expression in G1_1; (B) The FC of gene expression in G1_2; (C) Th FC of gene expression in G1_3; (D) The FC of gene expression in G1_4. The x-axis represents the name of sixteen candidate genes, y-axis shows the FC increase/decrease in expression level of the genes. **Fig. S2.** qRT-PCR validation of candidate genes in G2 group. (A) The fold change (FC) of gene expression in G2_1; (B) The FC of gene expression in G2_2; (C) Th FC of gene expression in G2_3; (D) The FC of gene expression in G2_4. The x-axis represents the name of sixteen candidate genes, y-axis shows the FC increase/decrease in expression level of the genes. **Fig. S3.** qRT-PCR validation of candidate genes in G3 group. (A) The fold change (FC) of gene expression in G3_1; (B) The FC of gene expression in G3_2; (C) Th FC of gene expression in G3_3; (D) The FC of gene expression in G3_4. The x-axis represents the name of sixteen candidate genes, y-axis shows the FC increase/decrease in expression level of the genes.

## Data Availability

All data supporting the conclusions of this article are included within the article and its additional files. The RNA-seq data presented in the study are deposited in the NCBI database Sequence Read Archive (SRA), accession number PRJNA981688.

## Abbreviations

BR: Brassinosteroid
CKXs: Cytokinin oxidase/dehydrogenases
DAF: Day after flowering
DEGs: Differentially expressed genes
DGAT: Diacylglycerol acyltransferase
DRTs: Domestication-related traits
EAR: Enoyl-ACP reductase
FAD: Omega-6 fatty acid desaturase
FC: Fold changes
FDR: False discovery rate
FPKM: Fragments per kilobase of transcript per million fragments mapped
GA2OX: Gibberellin-2-oxidase
GO: Gene ontology
GPAT: Glycerol-3-phosphate acyltransferase
HGW: Hundred-grain weight
KAS III: 3-Ketoacyl-ACP synthase III
KCS: 3-Ketoacyl-CoA synthase
KEGG: Kyoto Encyclopedia of Genes and Genomes
kME: Module Eigengene
LA: Linoleic acid
LACS: Long chain acyl-CoA synthetase
LNA: Linolenic acid
LPA: Lysophosphatidic acid
OA: Oleic acid
OC: Oil content
PA: Palmitic acid
PDCT: Phosphatidylcholine: diacylglycerol choline phosphotransferase
PDHC: Pyruvate dehydrogenase complex
PLC: Phospholipase C
PLD: Phospholipase D
PP2C: Phosphatase 2C
qRT-PCR: Quantitative real-time PCR
SA: Stearic acid
SAD: Stearoyl-ACP desaturase
SH: Seed height
SL: Seed length
SPB: Squamosa promoter binding protein
SW: Seed width
WGCNA: Weighted gene co-expression network analysis
