# Supplementary figures and images for: WGCNA and transcriptome profiling reveal hub genes for key development stage seed size/oil content between wild and cultivated soybean
Source: BMC Genomics. 2023 Aug 28;24:494. doi: 10.1186/s12864-023-09617-6 (PMC10463976; doi:10.1186/s12864-023-09617-6)

Fig. S1

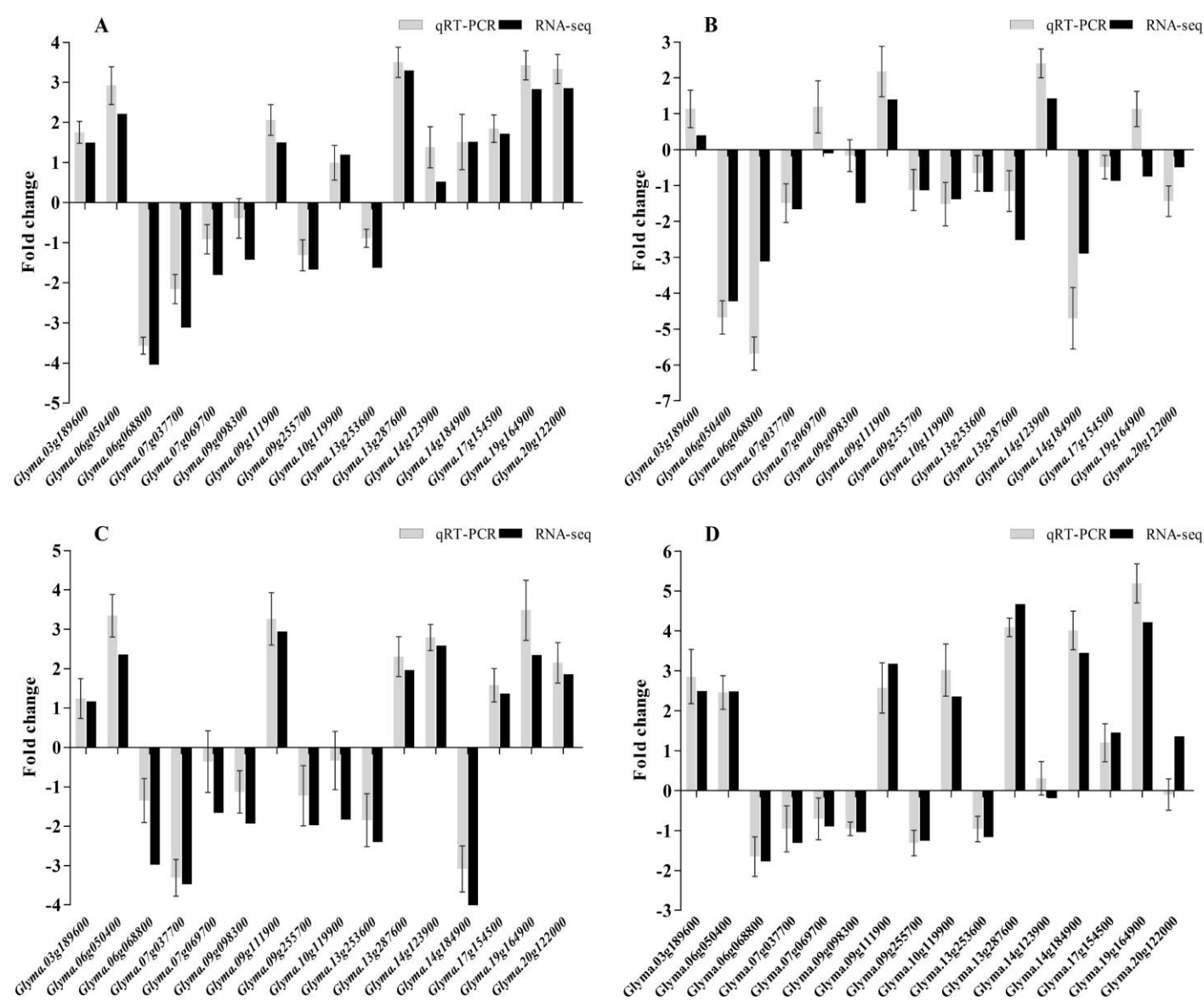

Fig. S2

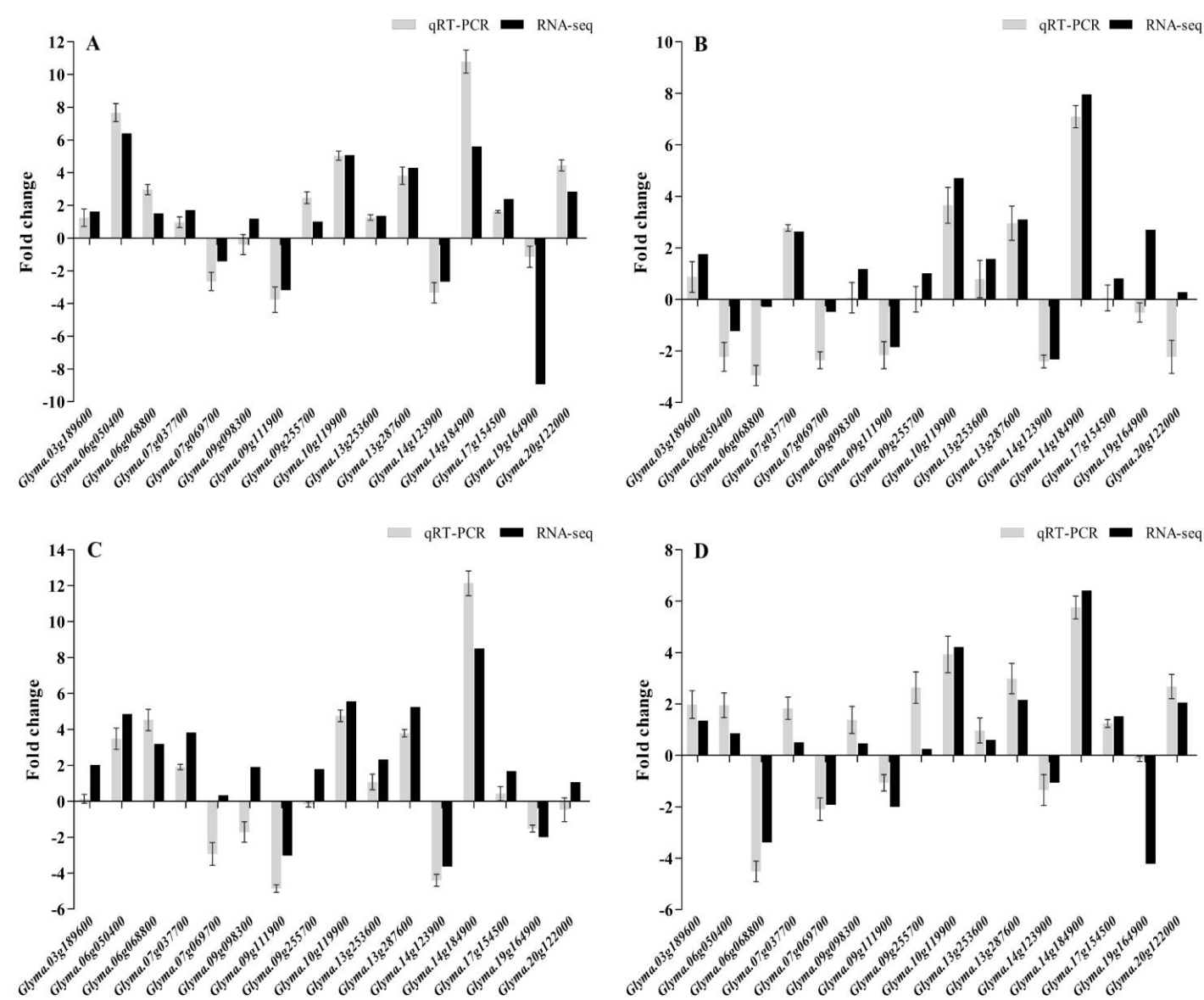

Fig. S3

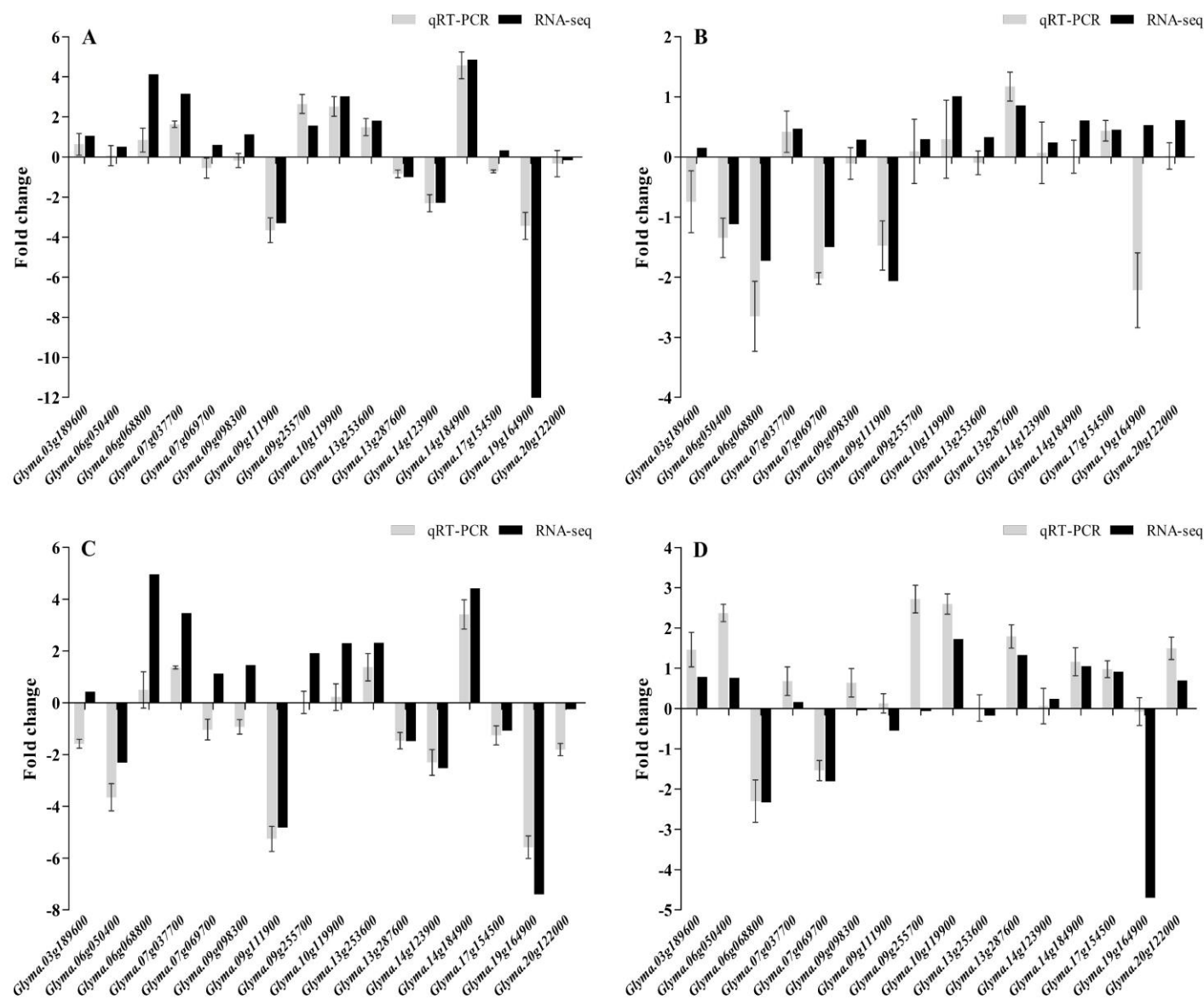

Supplement: Supplementary file 1 — Additional file 1: Table S1. Statistics of traits associated with soybean seed oil and seed weight for RNA-seq sample. SL: seed length; SW: seed width; SH: seed height; HGW: hundred-grain weight ; OC: oil content; OA: oleic acid; LNA: linolenic acid. The values associated with seed size and oil were continuously measured from 2015 to 2017. The data is presented in the form of mean ± standard deviation (SD). Table S2. Information of all primer sequence in qRT-PCR. Table S3. The candidate genes involved in lipid metabolism. Table S4. The candidate genes involved in regulation of seed size. Table S5. Candidate hub genes in six modules according to kME value. Fig. S1. qRT-PCR validation of candidate genes in G1 group. (A) The fold change (FC) of gene expression in G1_1; (B) The FC of gene expression in G1_2; (C) Th FC of gene expression in G1_3; (D) The FC of gene expression in G1_4. The x-axis represents the name of sixteen candidate genes, y-axis shows the FC increase/decrease in expression level of the genes. Fig. S2. qRT-PCR validation of candidate genes in G2 group. (A) The fold change (FC) of gene expression in G2_1; (B) The FC of gene expression in G2_2; (C) Th FC of gene expression in G2_3; (D) The FC of gene expression in G2_4. The x-axis represents the name of sixteen candidate genes, y-axis shows the FC increase/decrease in expression level of the genes. Fig. S3. qRT-PCR validation of candidate genes in G3 group. (A) The fold change (FC) of gene expression in G3_1; (B) The FC of gene expression in G3_2; (C) Th FC of gene expression in G3_3; (D) The FC of gene expression in G3_4. The x-axis represents the name of sixteen candidate genes, y-axis shows the FC increase/decrease in expression level of the genes. [file 12864_2023_9617_MOESM1_ESM.zip › Fig S1-S3.pdf]
